# A Review of Fluoride Removal from Phosphorous Gypsum: A Quantitative Analysis via a Machine Learning Approach

**DOI:** 10.3390/ma17143606

**Published:** 2024-07-22

**Authors:** Huagui Jin, Yixiao Wang, Xuebin An, Shizhao Wang, Yunshan Wang, Gang Yang, Lufang Shi, Yong Sun

**Affiliations:** 1 School of Chemical Engineering and Technology, Hebei University of Technology, Tianjin 300130, China; 18539657358@163.com (H.J.); shizhaow@163.com (S.W.); 2 Department of Chemical Engineering, University College London (UCL), Torrington Place, London WC1E 7JE, UK; yixiao.wang.22@ucl.ac.uk; 3 Institute of Process Engineering, Chinese Academy of Sciences, Beijing 100190, China; xban@ipe.ac.cn (X.A.); yanggang@ipe.ac.cn (G.Y.); 4 Each Energy Australia, James Ruse Drive, Paramatta, NSW 2116, Australia; thks-aries@hotmail.com; 5 Department of Chemical and Environmental Engineering, University of Nottingham Ningbo China, Ningbo 315100, China

**Keywords:** phosphogypsum, F removal, quantitative analysis, machine learning, review

## Abstract

This review comprehensively explores fluoride removal from phosphogypsum, focusing on its composition, fluorine-containing compounds, characterization methods, and defluorination techniques. It initially outlines the elemental composition of phosphogypsum prevalent in major production regions and infers the presence of fluorine compounds based on these constituents. The study highlights X-ray photoelectron spectroscopy (XPS) as a pivotal method for characterizing fluorine compounds, emphasizing its capability to determine precise binding energies essential for identifying various fluorine species. Additionally, the first-principle density functional theory (DFT) is employed to estimate binding energies of different fluorine-containing compounds. Significant correlations are observed between the total atomic energy of binary fluorides (e.g., of alkali metals, earth metals, and boron group metals) and XPS binding energies. However, for complex compounds like calcium fluorophosphate, correlations with the calculated average atomic total energy are less direct. The review categorizes defluorination methods applied to phosphogypsum as physical, chemical, thermal, and thermal-combined processes, respectively. It introduces neural network machine learning (ML) technology to quantitatively analyze and optimize reported defluorination strategies. Simulation results indicate potential optimizations based on quantitative analyses of process conditions reported in the literature. This review provides a systematic approach to understanding the phosphogypsum composition, fluorine speciation, analytical methodologies, and effective defluorination strategies. The attempts of adopting DFT simulation and quantitative analysis using ML in optimization underscore its potential and feasibility in advancing the industrial phosphogypsum defluorination process.

## 1. Introduction

A large amount of phosphorus-containing gypsum (around 70 million tons annually) generated in the production of wet-process phosphoric acid has become a major bottleneck restricting the sustainable development of phosphorous chemical enterprises [1]. How to effectively transform phosphorus-containing gypsum has become the focus of solid waste clean conversion [2]. Although scholars and the industry have made many technological breakthroughs in the transformation of phosphogypsum, such as building material preparation [3], CO_2_ mineralization [4], transesterification catalyst preparation [5], adsorbents [6] and other fields in recent years, there are still many issues that need systematic and in-depth research. Among these issues (such as impurity removal, calcium sulfate crystallization, calcium sulfate activation, calcium sulfate thermal reduction, etc.), the separation and removal of impurities in phosphorus gypsum are the most important [7]. Impurities not only significantly hinder the crystallization of calcium sulfate but also affect the subsequent transformation of calcium sulfate. The most important parameters in the existing impurity removal and purification pretreatment process are phosphorus (P) removal and fluorine (F) removal, with soluble phosphorus and fluorine less than 0.1 wt%, and the residual organic matter is removed at the same time. Phosphorus impurity removal attracts great attention [8], while the removal of fluorine impurities still needs more systematic and in-depth investigation. Taking the preparation of building materials with gypsum as an example, when the fluorine impurity content increases to about 1 wt%, the accompanying calcium fluoride crystals will seriously affect the growth of hemihydrate gypsum (β-CaSO_4_·0.5H_2_O) crystals and then significantly reduce the mechanical strength of hemihydrate gypsum [9,10,11,12]. In addition, the long-term accumulation of a large amount of fluoride in the environment, which can inhibit plant photosynthesis, damage plant tissue, reduce human bone density, cause abnormal protein metabolism, and significantly interfere with reproductive function, has increasingly attracted the attention of society and academia [13,14]. Unlike the reviews summarizing the achievements of works by scholars in the relevant fields, this article conducts a more in-depth quantitative analysis of defluorination operating conditions using a self-developed machine learning (ML) artificial neural network (ANN) algorithm based on TensorFlow. It performs quantitative analysis and simulation of relevant defluorination process parameters, further providing more insightful guidance of optimal parameter ranges. Moreover, this work attempts to estimate the total energy of fluorine-containing impurities in phosphogypsum based on the reported crystallite Miller index of crystallite fluorine-containing compounds by taking a density functional theory (DFT) approach, and correlates the variations in their binding energy obtained from X-ray photoelectron spectroscopy (XPS) characterization. A systematic review of fluorine-containing impurities in phosphogypsum, estimation of their total energy using the DFT approach, and quantitative analysis of defluorination processes using TensorFlow for ML, to the knowledge of authors, have not been reported before.

## 2. Theorical Work

### 2.1. Total Energy Calculation from Density Functional Theory (DFT) Approach

In this work, to produce more insightful information on the intrinsic properties (such as total energies) in fluorine compounds and their corresponding experimentally tested chemical shifts presented in literature reports, an estimation of binding energies using the DFT approach was deployed for certain fluorine compounds highly likely to be found. The total energy was estimated using the widely employed charge density function shown below [15,16]:

(1) $$E_{t}\left[\rho \right]=K\left[\rho \right]+U\left[\rho \right]+E_{XC}[\rho ]$$

where ρ is the density of the investigated molecular cluster (kg·m^−3^), $E_{t}\left[\rho \right]$ is the total energy of the system containing the investigated clusters of molecules (kJ·mol^−1^), $K\left[\rho \right]$ is dynamic energy (kJ·mol^−1^), $U\left[\rho \right]$ (kJ·mol^−1^) is the energy caused by Coulombic interactions, and $E_{XC}[\rho ]$ (kJ·mol^−1^) represents multiple molecular interactions within the system. Although a large discrepancy might be incurred by arbitrarily assuming that the crystallite structure is according to the reported Miller Indices of the compounds, the trend of these calculated values might be useful in the context of comparisons among different fluorine compounds.

### 2.2. Data Training and Analysis

**Figure 1:**
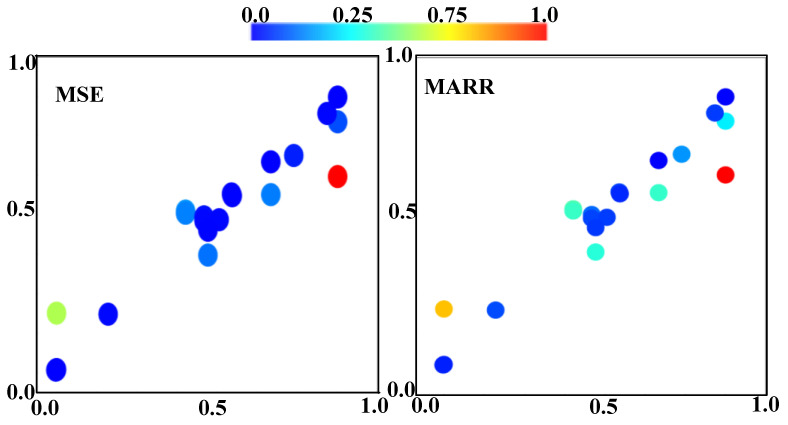
MSE and MARR results of literature reports and ML predictions used for validation.

In this work, random data selection together with a cross-validation approach were deployed. To evaluate the accuracy of data training, the mean square error (*MSE*) and mean average relative residual (*MARR*) were calculated as follows [17,18]:

(2) $$MSE\%=\frac{1}{N_{rep}}{\sum_{j=1}^{N_{exp}}{(d}_{i}^{rep}-d_{i}^{cal})}^{2}\times 100\%$$

(3) $$MARR\%=\frac{1}{N_{rep}}\sum_{j=1}^{N_{exp}}\left(\frac{\left|d_{i}^{rep}-d_{i}^{cal}\right|}{d_{i}^{rep}}\right)\times 100\%$$

where *N_rep_* is the number of literature-reported data (-), and $d_{i}^{rep}$ and $d_{i}^{cal}$ are data reported from references and calculations from ML algorithms. Acting as a crucial metric for evaluating the performance of the predictive models, the *MSE* is a good indicator of the quality of an estimator, while *MARR* serves as a good means of gauging uncertainties of statistical residues. In this work, the *MSE* and *MARR* analyses of all collected literature reports are summarized in Figure 1. The *MSE* presents less than 20%, indicating reasonably acceptable training outcomes of the investigated data.

As for the *MARR* results, most of the outcomes are within the reasonable ±25% range, indicating statistically trustworthy data predictions of the models in this work using TensorFlow. Please note that the increased numbers of datum generally will enhance the accuracy of model predictions. As more and more reported literature works in the relevant field accumulate, the accuracy will significantly improve.

### 2.3. Methodology of Machine Learning and Artificial Neural Networks (ANNs)

**Figure 2:**
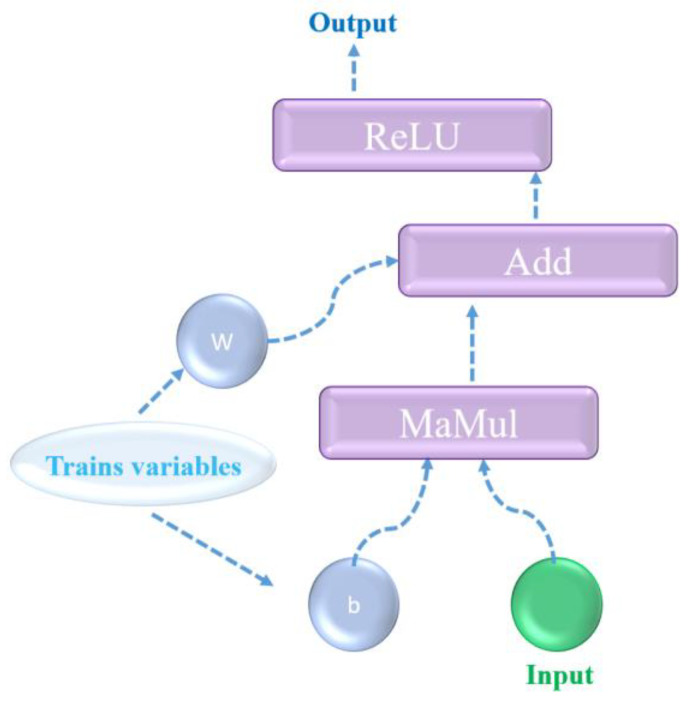
Illustration the workflow of TensorFlow, where w refers to weights and bias of linear models used in this simulation, MaMul performs matrix multiplication, Add refers to element-wise addition of two tensors, input refers to the collected parameters reported from the literature, and output refers to simulation results.

Machine learning (ML) is a branch of artificial intelligence that focuses on building systems that learn from data and improve their performance without being explicitly programmed. There are several important steps involved for ML in this work: (1) problem definition (we attempt to correlate the clusters of data with objective data), (2) data collection (in this work, data were collected and extracted from literature reports), (3) data preparation and treatment (in this work, all data were normalized for convenience of learning), and (4) model selection, as this is one the most critical parts of ML apart from the quality of the collected data. In this work, the multilayer perceptron (MLP) was used. For the MLP activation function, both linear and non-linear activation functions exist. The learning rate is arbitrarily set at 0.01 in this work to avoid drying issues during itineration. The entire ANN architecture is based on the use of TensorFlow, which is high level of application programming interface (API). A schematic diagram of the workflow of TensorFlow is shown in Figure 2, and the detailed configurations of hidden layers and their corresponding functions can be found in our reported works [19]. The Rectified Linear Unit (ReLU) is a common activation function used in neural networks to introduce non-linearity into the model. Before being fed into TensorFlow, the collected data points from the referenced literature were normalized to construct vectors. For instance, in the acid leaching learning process, parameters of operational temperature, duration, and solid–liquid ratio (SLR) were initially normalized using NumPy commands and automatically exported as a csv format in the default project folder. After preprocessing into a data matrix, the normalized data were saved as a training dataset for model prediction. To establish the hidden layers, a commonly used configuration with five hidden layers was employed, using ReLU as the activation function. Default settings were used for both weight and bias parameters during the training process in TensorFlow. The keras API was imported in the main function, which possesses both user-friendly and powerful capability features for developing various types of neural networks. (5) Data training strategy: in this work, due to the limited number of training datasets, we adopted a cross-validation approach to maximize their use. (6) Monitoring and data reporting: in this work, the epoch and learning rate were arbitrarily set at 10,000 and 0.001, respectively, with a reasonable uncertainty of ±25% set as the default. The simulated outputs were plotted using the matplolib library in Python.

## 3. Results

### 3.1. Composition of Phosphogypsum in Different Regions and Different Potentially Fluorine-Containing Compounds

China is vast in territory, and the key producing areas of phosphogypsum are unevenly distributed in provinces such as Yunnan, Hubei, Guizhou, Sichuan, Anhui, and Shandong. Table 1 summarizes the main components and corresponding fluorine contents of phosphogypsum in different provinces. First, the fluorine content shows significant differences with different regions [20]. For instance, the fluorine content in phosphogypsum from some provinces (e.g., Yichang, Hubei, with a-F 2.51 wt%) is comparatively higher than that in other provinces (e.g., Sichuan and Shandong, where the fluorine content is extremely low at <1 wt%). Overall, except for individual provinces like Hubei, Sichuan, and Yunnan, where the fluorine content of phosphogypsum ranges from 1 to 3 wt%, the fluorine content in other provinces is below 1 wt%. This means that the defluorination process of phosphogypsum must consider the origin and source of the phosphogypsum. Second, compared to the P—phosphor element, the content of F—fluoride in phosphogypsum is relatively low. Existing data show that the P/F mass ratio usually fluctuates between 0.7 and 9, indicating significant regional differences in the samples. Meanwhile, the technical difficulty of removing relatively low concentrations of F elements will become relatively high as the energy consumption and chemical intensity generally are inversely proportional to the level of impurity in the phosphogypsum matrix.

Taking the fluorapatite (Ca_5_(PO_4_)_3_F), the main component of phosphate ore, as an example, through acid leaching of phosphate ore using the dihydrate wet process or a ternary system (H_2_O-H_2_SO_4_-H_3_PO_4_), the F elements bound in different forms in the phosphate ore are dissolved by strong acids. The main chemical reactions are briefly summarized as follows:

(4) $${Ca}_{5}{\left({PO}_{4}\right)}_{3}F+7H^{+}=3H_{2}{PO}_{4}^{-}+HF↑+5{Ca}^{2+}$$

(5) $$6HF+{SiO}_{2}=H_{2}{SiF}_{6}+2H_{2}O$$

(6) $$2H_{2}{SiF}_{6}+{SiO}_{2}=3{SiF}_{4}↑+2H_{2}O$$

During the acid leaching process of the phosphogypsum, certain F ions will become HF gas (Equation (4)) and fluorosilicic acid (Equation (5)), respectively. However, due to the instability of fluorosilicic acid, it will further react with the leached silicon dioxide (SiO_2_) to produce gaseous silicon tetrafluoride (Equation (6)). Other F ions will combine with cations (such as H^+^, Na^+^, K^+^, Mg^2+^, Ca^2+^, Al^3+^, etc.) in solution. During the reaction between phosphate ore and sulfuric acid to produce phosphoric acid, the reaction rate is relatively fast, resulting in an environment where the F^−^ anions and cations are mainly present in a solution system dominated by the existence of phosphoric acid (H_3_PO_4_) [37]. Additionally, in the presence of multiple cations, especially when Al^3+^ ions are present, different complexes will be formed under different pH conditions. For example, when the pH is between 3 and 7, in a solution system containing cations (such as Ca^2+^, Mg^2+^, and Al^3+^) and dominated by phosphorous acid (H_3_PO_3_), besides the formation of calcium fluoride (CaF_2_) precipitates, Al^3+^ can promote the decomposition of SiF_6_^2−^ to generate Al-F complexes. However, when the pH is less than 3, Al^3+^ is more likely to directly combine with free F^−^ to form Al-F complexes [38]. The stability of Al-F complexes, in descending order, is AlF^4−^, AlF^2+^, AlF_5_^2−^, AlF^2+^, AlF_3_, and AlF_6_^3−^. Yet, as the ratio of F to Al increases, Al-F complexes are more likely to exist in the form of complexes with a higher F/Al ratio [39,40]. In addition to binary fluorine compounds, there are also multi-component fluorine compounds present in the solution system. Based on the main possible elements in the phosphate ore, a summary of the main fluorine compounds is presented in Table 2.

From the perspective of solubility, fluorine-containing compounds in phosphogypsum can be divided into two major categories: the insoluble and the soluble (sodium floride—NaF, potassium fluoride—KF) compounds. For insoluble fluorine-containing compounds, the solubility product constants (K_sp_) exhibit different orders of magnitude, ranging from 10^−3^ for sodium fluorosilicate (Na_2_SiF_6_) to 10^−61^ for fluorapatite (Ca_5_(PO_4_)_3_F). The differences in solubility provide an important thermodynamic basis for the separation of fluorides based on phase equilibrium methods, such as precipitation.

### 3.2. Characterization Approaches for Fluorine-Containing Compounds in Phosphogypsum

**Figure 3:**
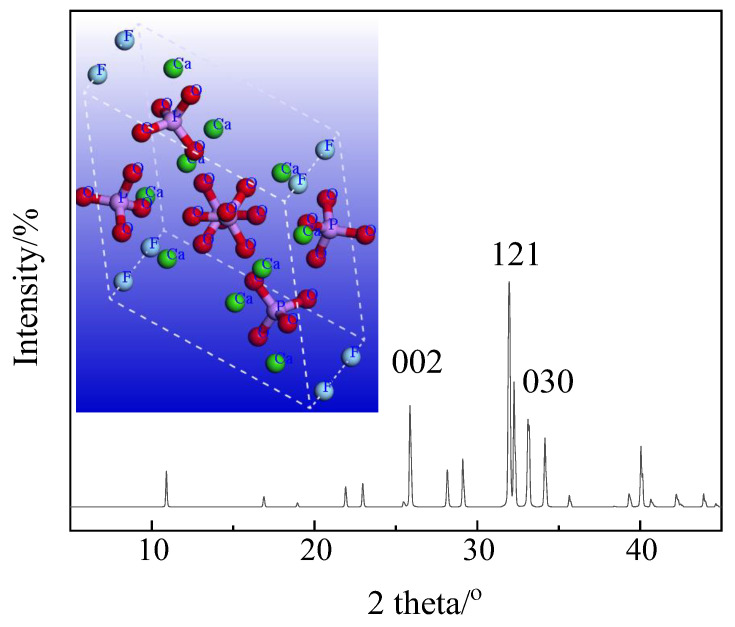
Calculated XRD spectrum of fluoroapatite (Ca_5_(PO_4_)_3_F).

Characterization is an important means to deeply understand the enrichment forms of fluorides in phosphogypsum. The variety of measurement methods include X-ray absorption spectroscopy (XAS) [44], nuclear magnetic resonance spectroscopy (NMR) [45], X-ray photoelectron spectroscopy (XPS) [46], X-ray diffraction (XRD) [47], Fourier transform infrared spectroscopy (FTIR) [48], etc. Currently, commonly used and economical characterization methods include XPS, XRD, FTIR, etc. Taking XPS as an example, photoelectron spectroscopy reveals the chemical shift caused by the difference in the chemical environment of the inner electrons of atoms. Due to the different Coulomb forces and electron shielding effects between atoms within different fluorine-containing compounds, F1s exhibits different binding energies in fluorine-containing compounds. The reported values of the binding energy of F1s in fluorine-containing compounds in phosphogypsum vary roughly between 683.9 eV (NaF) and 688 eV (AlF_3_). In order to further understand the variation in chemical shifts caused by the difference in the chemical environment of the inner electrons of fluorine atoms from the first-principle perspective, this article attempts to construct the crystal structure of the corresponding compounds based on the elemental composition (Table 2) and the lattice constants of existing related possible crystallite lattice parameters (Table 3—Miller Indices). When adjusting and optimizing the coordination of molecular structures, the total energies (kcal/mol/atom) of constructed molecules could be estimated by using force fields via the DFT approach. The detailed parameter and simulation settings can be found from our published work [49,50]. To demonstrate how we calculate the total energy, we take the complex compound of calcium fluoroapatite (Ca_5_(PO_4_)_3_F) as an example in showing the process of how to construct coordinates of molecules and how to validate the constructed structure of molecules by comparing its simulated XRD spectrum with the reported Miller Index of fluoroapatite. The input coordinates of fluoroapatite are summarized in supplementary Table S1. The estimated XRD spectrum of fluoroapatite is shown in Figure 3.

The calculated feature peaks generally agree well with the reported values of the Miller Index of fluoroapatite (Table 3), which indicates reasonably constructed coordination (Table S2) is appropriate for total energy estimation. Thus, using the same methodology, the total energies of all possible fluoride compounds (Table 3) could be estimated accordingly. The chemical shifts (eV) of compounds from reported XPS characterization and the corresponding DFT-calculated average atomic total energies (kcal/mol/atom) are summarized in Table 4.

From the perspective of binding energy, binary compounds formed by fluorine and alkali metals (Na, K) exhibit lower chemical shifts, and their corresponding average atomic total energies also show lower values. Additionally, larger atomic radii correspond to higher fluorine chemical shifts and average atomic total energies, showing a positive correlation. Alkaline earth metals (Mg, Ca) tend to lose two exterior electrons easily, resulting in higher chemical shifts and average atomic total energies compared to alkali metals. The atomic radius of alkaline earth metals is positively correlated with their corresponding fluorine chemical shifts and average atomic total energies. Boron group elements (such as aluminum—Al) typically release their outermost three electrons (p1 orbit) relatively easily, thus exhibiting higher fluorine chemical shifts and average atomic total energies. The reported fluorine chemical shifts show good consistency with the values from DFT calculation. For multi-component salts, due to their complex crystal structure and overlapping crystal peaks with other crystal fingerprint peaks, neither the reported fluorine chemical shifts nor the DFT-calculated average atomic total energies exhibit clear trends or correlations. Among the multi-component salts, potassium fluosilicate (K_2_SiF_6_) exhibits the largest fluorine chemical shift (687 eV) and a relatively high average atomic total energy (51.625 kcal/mol/atom). On the other hand, sodium fluoroaluminate (Na_3_AlF_6_) shows the lowest calculated average atomic total energy (13.948 kcal/mol/atom), but its corresponding fluorine chemical shift (686.38 eV) is not the lowest among the multi-component salts. This may be attributed to the fact that the multiple outer electrons of boron group elements are better at stabilizing the internal energy of the crystal compared to semiconductor elements (such as silicon), resulting in lower average atomic total energies from theoretical calculations. However, reported literature values of fluorine chemical shifts are often influenced by impurities and multiple compounds, which adds challenges to theoretical interpretation. In summary, considering the variability in fluorine-containing compositions in phosphogypsum after acid leaching and crystallization due to different phosphate rock origins, this paper systematically reviews the major fluorine-containing compounds in phosphogypsum, along with their commonly used characterization methods and fingerprint information. Furthermore, DFT calculations reveal a strong correlation between XPS binding energy (eV) and average atomic total energy (kcal/mol/atom) for binary fluorine-containing compounds (alkali metals, alkaline earth metals, and boron group metals). However, for complex compounds such as sodium fluoroaluminate (Na_3_AlF_6_) and calcium fluoroapatite (Ca_5_(PO_4_)_3_F), the theoretical calculations show lower average atomic total energies. Calculation of total energies of fluorine-containing compounds and construction of crystallite atomic structures (with coordinates after validation) lay a theoretical foundation and guidance for a subsequent F removal mechanism during separation for future works.

### 3.3. Summary of Separation Methods and Their Corresponding Quantitative Analysis

#### 3.3.1. Physical Approach

**Figure 4:**
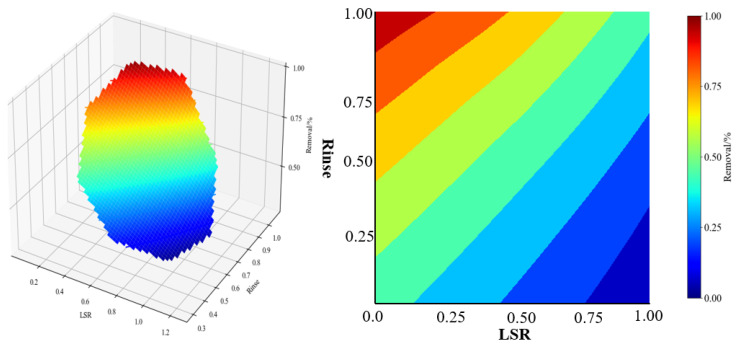
ML results for impacts of the liquid–solid ratio (LSR) and number of washes (Rinse) on defluorination efficiency.

When it comes to the physical treatment of PG (phosphogypsum), this typically refers to the separation of fluorine impurities through physical means such as water washing and sieving. The advantage lies in its relatively low cost, especially for soluble fluorides like sodium fluoride and potassium fluoride, but its effectiveness in treating insoluble fluorides like calcium fluoride and fluoroapatite is limited. Among the physical methods, water washing is a common and simple method for defluorination, and important operational parameters include the liquid–solid ratio and the number of washes. Table 5 summarizes some literature reports and corresponding defluorination results. To derive more valuable trend results, this paper utilizes TensorFlow to build our own neural network for machine learning. All machine learning processes are conducted using TensorFlow in Python with 10,000 as the default iterations [49,80]. A simulation plot with approximately ±25% uncertainty was drawn. The simulation results are shown in Figure 4. Clearly, the number of washings primarily determines the extent of F removal, more so than the liquid-to-solid ratio (LSR).

With further improvement of the water washing process, Fang Zhukun et al. developed a new highly efficient water washing process with low water consumption, which primarily reduces water consumption to 0.5 tons of water per ton of phosphogypsum by increasing the temperature of the washing water and effective recycling, achieving a defluorination rate of less than 0.05% [87]. While the appealing feature is the effective removal of soluble fluorides from calcium sulfate dihydrate [88], its weaknesses are the following: (a) it consumes a significant amount of water; (b) it poses a potential ecological threat to water bodies, leading to secondary pollution; and (c) its ability to remove insoluble fluoride compounds is limited. Therefore, the water washing method has its intrinsic limitations.

**Figure 5:**
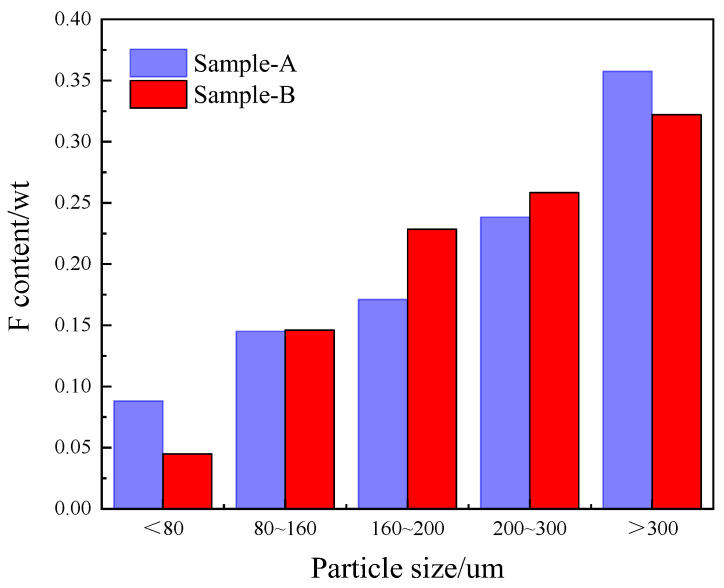
Particle size and corresponding fluorine contents, in Samples A and B of phosphogypsum, where A is reported from the literature [89] and B is reported from the literature [90].

The screening method can separate a portion of fluorine-containing compounds based on the different fluorine compounds present in phosphogypsum with different particle sizes. By screening different particle sizes, a separation of fluorine-containing compounds can be achieved. Figure 5 shows the particle size distributions and the corresponding fluorine compound contents reported in two sets of the literature.

Generally, phosphogypsum particles with a diameter above 300 μm still contain approximately 0.3% of fluorine compounds. As the particle size of phosphogypsum decreases, the fluorine compound content also decreases accordingly, indicating that smaller phosphogypsum particles are less likely to entrap and embed fluorine compounds during the crystallization process [91]. Therefore, the smaller phosphogypsum particles (<80 μm) will result in a relatively lower fluorine content in the phosphogypsum. However, the physical screening method cannot effectively separate fluorine compounds from a large amount of larger phosphogypsum particles.

#### 3.3.2. Chemical Approach

**Figure 6:**
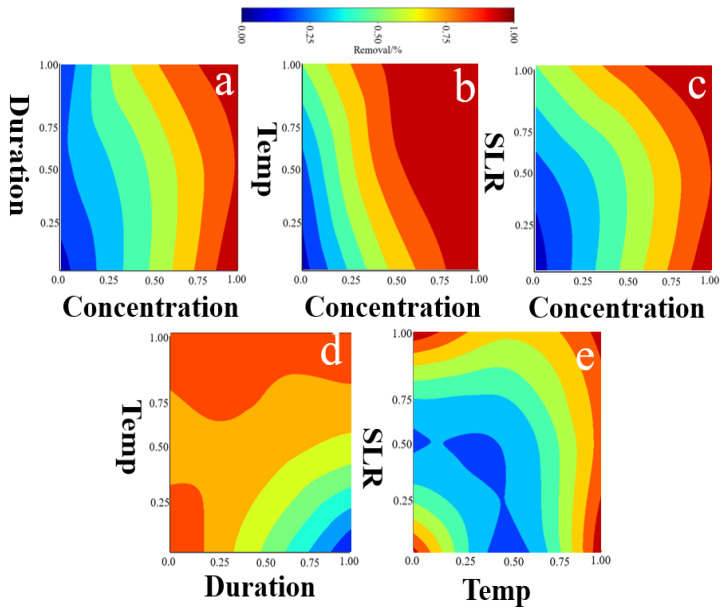
The ML results of binary parameters upon F removal, where (**a**) refers to concentration vs. time, (**b**) refers to concentration vs. temp., (**c**) refers to concentration vs. SLR, (**d**) refers to time vs. temp., and (**e**) refers to temp. vs. SLR.

The chemical approach involves adding alkaline or acidic substances to react with impurities in phosphogypsum, converting them into inert or soluble substances. However, this method is not particularly effective in removing organic substances [92]. Based on the nature of the chemical substances added, the chemical methods are divided into alkaline contacting and acid treatment. Acid treatment involves the extraction of phosphogypsum with acid, dissolving fluorine impurities into the acid solution, thereby improving the quality of the phosphogypsum [93]. The typical use of sulfuric acid in the acid treatment of phosphogypsum and the corresponding changes in fluorine content (Table 6) as well as the influence of binary operational variable changes on fluorine removal are illustrated in Figure 6.

The impact of the acid concentration and duration is shown in Figure 6a. The higher F removal locates at high-end range of concentrations regardless of duration. This indicates that the sulfuric acid strength predominantly determines the F removal when comparing that with duration. The impact of acid concentration and reaction temperature is shown in Figure 6b. The pattern of (concentration vs. temperature) F removal appears slightly different. The highest F removal region sits in the top right-hand corner. This indicates that both concentration and temperature tend to yield synergistic impacts upon F removal with acid strength more weighted. The impact of acid concentration and SLR is shown in Figure 6c. Apparently, the sulfuric acid strength predominantly determines the F removal when comparing that with SLR. The impact of duration and reaction temperature is shown in Figure 6d. There are two main regions with relatively higher F removal; the first region sits in the left bottom corner and the second is located in the top range in regard to temperature. Clearly, it is reasonable to reject the first region and accept the second region. Thus, it is found that temperature tends to produce more impacts upon F removal when comparing that with duration. Indeed, a sufficient temperature (over 70 °C) is required. The impact of SLR and reaction temperature is shown in Figure 6e. Although four corner regions with high F removal show up, it is reasonable to accept the right-hand set bottom corner (indicating a higher temperature and lower SLR are needed to ensure better dissolution and mass transfer), while reject all other corner regions. The analysis of variance (ANOVA) shows that among the four operational variables, acid concentration is the most important statistical factor influencing defluorination of phosphogypsum. To achieve a defluorination rate of over 90%, the acid concentration needs to be controlled at above 45%. However, if there is a need to reduce the acid concentration, in order to achieve a defluorination rate of over 90%, the reaction temperature needs to be increased accordingly. For example, when the acid concentration decreases to 25%, to achieve a defluorination rate >90%, the corresponding reaction temperature needs to be increased to above 90 °C, as shown in the simulation results in Figure 6b. For the optimization of parameters such as reaction time and solid–liquid ratio, a more effective strategy is to first determine the acid concentration and reaction temperature, and then determine these two relatively minor statistical factors. By using an ML neural network model, basic optimization trends can be obtained even with very limited amounts of input data. These quantified simulation results provide very meaningful quantitative guidance for actual production optimization. In addition to using strong sulfuric acid, scholars have also reported the use of citric acid. The range of operational condition variations in these types of studies is relatively narrow. Therefore, for the use of citric acid in phosphogypsum defluorination, achieving good defluorination rates is generally possible when conditions are adroitly managed within the following range (concentration 40%, reaction time 25 min, and reaction temperature 30 °C) [101,102,103,104].

**Figure 7:**
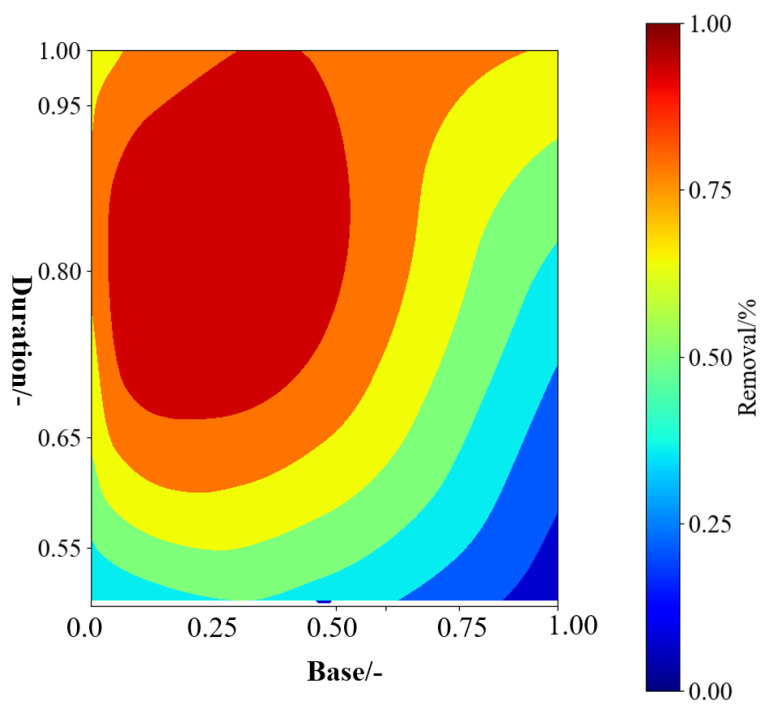
Simulation results of F removal with variations in base (calcium oxide—CaO) and duration from ML.

In addition to the acid method, the alkaline method is also a commonly used chemical approach. Alkaline treatment involves using alkaline substances to treat phosphogypsum, leading to the formation of insoluble or soluble fluorides in the liquid phase, thereby achieving the goal of fluoride removal from phosphogypsum. Alkaline substances typically used include lime (CaO) or ammonia (NH_3_). The employment of CaO and its corresponding operational conditions for F removal is shown in Table 7. From the simulated results (Figure 7), it can be observed that the amount of alkali added and the reaction time exhibit a synergistic effect, resulting in the optimal defluorination rate. When combining limited literature reports and simulation results, the optimal operating range for chemical defluorination using lime is as follows: alkali dosage of 3–5 wt% and reaction time of 16–18 h.

Apart from the use of lime, ammonia water can also be used as a reactant to leach F out of phosphogypsum. The operational range reported in the literature usually focuses on an ammonia concentration of around 10 wt%, a reaction time of 24 h, and a resultant fluorine removal rate that can reach round 60%. Therefore, this article suggests that the ideal operating conditions for defluorination of phosphogypsum using ammonia water should also fall within this range [106,107].

In summary, broadly speaking, two types of chemical approaches, namely acid and base treatment are widely adopted for F removal from phosphogypsum. For acid treatment, operational parameters such as acid concentration, temperature, and duration were found to be significant for F removal. In addition, as the concentration of sulfuric acid plays crucial role in crystallite transformation during precipitation and crystallization of gypsum, a relatively higher sulfuric acid concentration is preferable to achieve phosphogypsum with low impurities. Moreover, more ecofriendly weak acids such as citric acid were also reported to leach the F impurities from phosphogypsum. A weak strength of acid subsequently results in multiple acid/water rinses, which significantly increases the demand for wastewater treatment overall and accounts for its higher cost when compared with sulfuric acid with the same acidic strength. All the above limit the potential of citric acid (C_6_H_8_O_7_) treatment for F removal, which leaves sulfuric acid as the most widely and predominately used acid treatment reagent for F removal from phosphogypsum. Regarding alkali treatment, lime treatment was reported to only be effective to reduce the content of soluble fluorides. Moreover, the downstream process of using alkaline for F removal from phosphogypsum is reported to have longer and more complex procedures when compared with acid treatment.

#### 3.3.3. Thermal and Combined Approach

**Figure 8:**
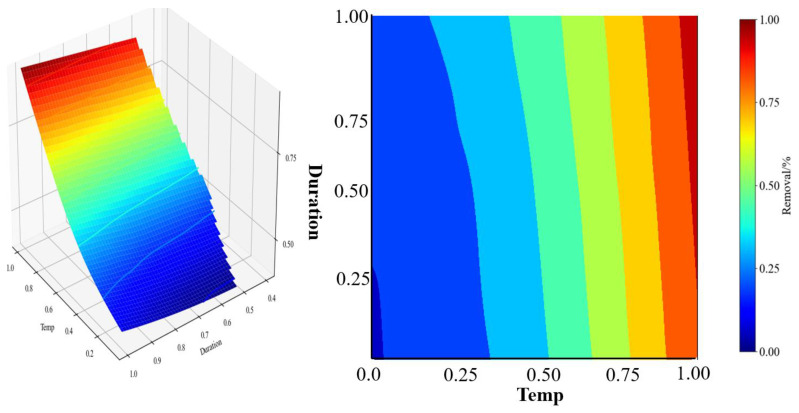
Simulation results of F removal with variations in temp. and duration from ML during thermal treatment.

The thermal treatment method involves the transformation or decomposition of fluorides in phosphogypsum under high-temperature conditions, thereby reducing the fluorine content in phosphogypsum and subsequently mitigating the impact of fluorine on its properties. Compared to the aforementioned treatment methods, the thermal method is more effective in removing insoluble fluorides (such as calcium fluoride—CaF_2_, fluoroapatite—Ca_5_(PO_4_)_3_F, etc.) from phosphogypsum [71]. During the thermal treatment, the volatile and soluble F are first removed from the matrix of phosphogypsum. For instance, as the temperature reaches 300 °C, the fluorosilicate will decompose into soluble and even vapor-phase fluoride compounds, i.e., potassium fluoride—KF_(S)_, sodium fluoride—NaF_(S)_, and silicon fluoride—SiF_4(g)_. With an increase in temperature during thermal treatment above 600 °C, the liberated soluble F (such as from sodium fluoride—NaF) will further react with calcium sulfate—CaSO_4_ to form insoluble F (such as calcium fluoride—CaF_2_). Due to its thermodynamic endothermic feature, the further increased temperature will be favorable in the formation of calcium fluoride [108]. It needs to be addressed that keeping the thermal treatment below 300 °C is not sufficient for F removal; thus, thermal treatment with a higher temperature is often practiced even though some insoluble and difficult-to-remove fluorides (calcium fluoride) are inevitably formed. The impacts of operational parameters when using thermal treatment upon F removal and the simulation results when adopting thermal treatment are shown in Table 8 and Figure 7, respectively. The simulation results (Figure 8) show that, to achieve a higher removal rate of insoluble fluorine (>90%), the optimal area is located in the top righthand-side corner, which indicates that the reaction temperature needs to be maintained at above 800–850 °C and the reaction duration needs to be kept at 2–3 h or even longer. Despite the higher temperature that the thermal treatment must adopt, its intrinsic advantages such as an absence of water washing, effective removal for both soluble and insoluble F, and much simpler downstream separation process all indicate its appealing benefits in F removal. To offset its main drawback of relatively higher energy consumption, this thermal treatment could be combined with other types of F removal approaches to further reduce energy consumption and produce the added value of phosphogypsum products [109].

Besides the conventional above-mentioned thermal treatment methods, many scholars have found that steam can promote the hydrolysis of halogen compounds, which is known as hydrothermal decomposition or high-temperature hydrolysis [110,111,112,113]. Under high temperature, as water molecules adsorb on the surface of fluorides, the energized water molecules can turn into radicals, i.e., O^2−^ or OH^−^, in association with fluoride compounds on the surface of phosphogypsum. By replacing the F^-^, volatile substances such as hydrogen fluoride—HF, hydroxyfluorides—FH_3_O, and oxides are formed [114]. Among the binary fluorides in phosphogypsum, the order of hydrolysis sensitivity from strong to weak follows aluminum fluoride—AlF_3_, magnesium fluoride—MgF_2_, calcium fluoride—CaF_2_, sodium fluoride—NaF, and potassium fluoride—KF [115]. Comparison with DFT calculations of the total energy of fluoride compounds (Table 4) suggests that the compounds that possess higher total energy tend to be reaction sensitive during high-temperature thermal treatment. As thermal treatment proceeds, however, the soluble fluorides (such as sodium fluoride and potassium fluoride) are prone to converting with calcium sulphate—CaSO_4_ into insoluble fluorides (such as calcium fluoride). For fluorine compounds, such as potassium hexafluorosilicate (K_2_SiF_6_), the steam will facilitate conversion from K_2_SiF_6_ to the formation of potassium silicate (K_2_SiO_3_) and hydrogen fluoride (HF) [116]. To further remove calcium fluoride from phosphogypsum, a relatively harsh temperature at least from 830 ± 10 °C is needed to convert calcium fluoride into gaseous hydrogen fluoride and solid calcium oxide, respectively [117].

**Table 8:** The impact of thermal treatment operational parameters upon insoluble F removal, where * refers to the values that have not been reported and the mean value of the array adopted to smooth the discrete matrix.

Temp/°C	Duration/h	Insoluble F Removal/%	Ref.
700	1	64.97	[118]
800	3	78.54	[10]
850 *	2 *	85.27	[26]
950	3	93.89	[119]

**Figure 9:**
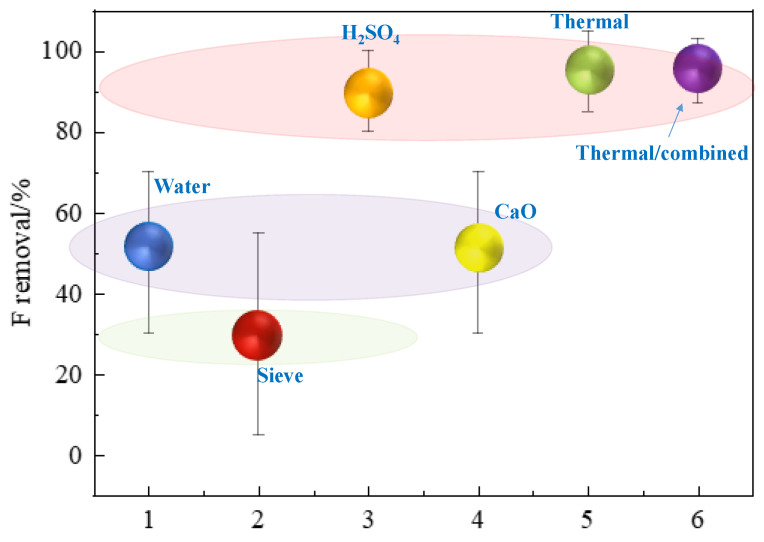
Comparison of the main F removal approaches, where Water refers to rinsing using water, Sieve refers to sieve screening, H_2_SO_4_ refers to sulfuric acid leaching, CaO refers to limestone or calcium oxide treatment, Thermal refers to thermal treatment, and Thermal/combined refers to combined thermal treatment.

Among the investigated approaches for F removal, their performance behave differently. For the convenience of discussion, herein, only six different types of approaches are compared and summarized in Figure 9, which are water, sieving, H_2_SO_4_, CaO, thermal, and thermal/combined. The performance of F removal can be broadly divided into three bands. The first-band approaches are the thermal, thermal/combined, and acid leaching (using sulfuric acid). This first band was found to be most effective for both soluble and insoluble F impurities’ removal from phosphogypsum with over a 90% removal rate. Yet, this high performance was compromised by the harsh operational conditions such as a higher temperature (over 800 °C), employment of steam, and higher acid strength (>40%).

The second-band approaches are water rinsing and limestone treatment. These approaches are more effective at soluble F removal from phosphogypsum. In addition, depending on origins of phosphorous ore and the types of F-containing compounds, the F removal performance varies significantly. The third-band approach is sieving screening; despite its poor F removal performance (less than 30% depending on particle size), its much lower environmental footprint (less wet chemicals consumed during separation) and feasibility for both soluble and insoluble F impurities’ removal show its merit as a supplementary approach.

## 4. Reflection and Future Opportunities

While significant experimental progress has been made in fluoride removal from phosphogypsum, challenges, i.e., tailoring the datum matrix, cleaning the data, optimizing the ML strategy, etc., still persist. Firstly, the heterogeneous nature of data from various sources, formats, and qualities underscores the critical role of data preprocessing in artificial intelligence (AI) technologies. In this study, for instance, meticulous selection and refinement of raw data were pivotal to extracting meaningful patterns for optimizing each specific separation method, thereby ensuring the accuracy of predictions. Secondly, the absence of a standardized format for reporting experimental data hinders the creation of a universal databank, necessitating extensive data cleaning before inputting into machine learning (ML) algorithms. This lack of uniformity complicates the integration and comparison of results across different literature reports. Thirdly, the future significance of open-source databases and software tools is anticipated to grow, making the Python (2021.2) programming language increasingly attractive for ML applications in this research realm. Consequently, sharing Python code is poised to become a prevalent practice in scholarly publications. Lastly, bridging the gap between microscale (e.g., DFT studies on a molecular level) and macroscale (characterization based on bulk analysis) investigations represents a persistent challenge. In this study, for example, estimating DFT total energies and establishing molecular spatial coordinates proved instrumental in enhancing our understanding of fluoride compound impurities within phosphogypsum structures. In summary, addressing these challenges—through enhanced data preprocessing, standardized data reporting practices, utilization of open-source tools like Python for ML, and integrating microscale and macroscale analyses—will facilitate advancements in fluoride removal strategies from phosphogypsum. Moreover, with the advancement of computational power, it will become an option to construct molecular cluster structures of phosphogypsum sandwiched with fluoride impurities (sodium fluoride—NaF, potassium fluoride—KF, calcium fluoride—CaF_2_, aluminum fluoride—AlF_3_, fluoroapatite—Ca_5_(PO_4_)_3_F, etc.); thus, in situ molecular simulation during the F removal process will become feasible in the foreseeable future.

## 5. Conclusions

Due to differences in the origins of phosphorous ores, the F impurities in phosphogypsum obtained by corresponding wet processes vary significantly. This review systematically analyzed and summarized the main potential elements of phosphogypsum from the major phosphate-ore-producing areas in China; thus, the possible fluorine-containing compounds in phosphogypsum were summarized. With the aid of the literature report from the XRD Miller Index, the crystallite molecular coordinates and structures of fluorine-containing compounds were constructed and optimized using the DFT approach. The total energies of fluorine-containing compounds were correlated with the variations in the binding energy obtained from XPS characterization. The results show that the total atomic energy of binary fluorides, i.e., alkali metals, alkaline earth metals, and boron group metals, has a positive correlation with XPS binding energy. For more complex multi-fluorine compounds (such as calcium fluorophosphate), the changing pattern between the XPS binding energy and the calculated total atomic energy is not obvious. The approaches of F impurity removal from phosphogypsum were categorized into three different types: physical, chemical, and thermal. The ML algorithm based on TensorFlow was used for quantitative analysis of these removal techniques, and the potential optimal conditions were obtained. The simulation results of the water washing method show that the number of water-washing rounds has a more significant impact on the removal of fluorine when compared with the liquid–solid ratio. To achieve a defluorination efficiency of over 50%, the number of water washing rounds needs to be maintained at least at four to five, and the liquid–solid ratio can be maintained at between three and four. The acid leaching method (sulfuric acid) shows that a more effective strategy is to first determine the acid concentration and reaction temperature, and then to determine the two relatively minor statistical variation factors (reaction duration and solid–liquid ratio). There is an optimal operating range for chemical defluorination using lime, namely, an alkali dosage of 3–5 wt% and reaction time of 16–18 h. For the removal of insoluble fluorine in phosphogypsum, the thermal method is more effective. The quantitative simulation results show that to achieve a higher removal rate of insoluble fluorine (>90%), the reaction temperature needs to be controlled at above 800–850 °C and the reaction time also needs to be more than 2–3 h. In summary, the use of machine learning algorithms based on neural networks will provide qualitative and quantitative optimization references for different separation processes, which provides valuable guidance for the optimization of subsequent processes.

## Figures and Tables

**Table 1:** Composition and fluorine content of typical phosphogypsum from different provinces. “-” means that the information is not reported in the literature or the content is below the detection limit, where ^a^, ^b^ and ^c^ refers to different samples that were collected from the same province.

Region	CaSO_4_	SiO_2_	P_2_O_5_	K_2_O	Al_2_O_3_	Na_2_O	Fe_2_O_3_	MgO	F	P/F	Ref.
Yunnan ^a^	60.97	11.74	0.35	0.27	0.08	-	0.15	-	0.16	2.19	[21]
Yunnan ^b^	70.68	9.43	2.04	-	0.24	-	0.14	0.06	0.52	3.92	[22]
Yunnan Honghe	71.15	7.02	3.10	0.14	0.58	0.74	0.15	0.47	1.17	2.65	[23]
Hubei ^a^	63.02	13.82	2.16	0.39	0.86	0.48	0.38	0.15	0.73	2.96	[24]
Hubei ^b^	67.38	15.60	0.93	0.61	0.47	0.20	0.38	0.01	0.76	1.22	[24]
Hubei ^c^	74.21	12.24	0.94	0.03	0.00	0.10	0.20	0.05	0.95	0.99	[24]
Hubei Yichang ^a^	64.18	5.91	6.26	-	1.18	-	1.49	0.64	2.51	2.49	[25]
Hubei Yichang ^b^	71.00	5.32	0.80	0.24	0.28	0.22	0.20	-	0.99	0.81	[26]
Hubei Xingfa	86.82	8.01	1.31	-	0.29	0.34	0.56	0.12	1.71	0.77	[27]
Guizhou banshui	81.60	5.79	1.66	0.14	0.60	0.74	0.15	0.21	0.79	2.10	[28]
Guizhou	73.85	4.17	1.24	0.14	0.19	0.74	0.15	0.16	0.35	3.54	[23]
Sichuan	72.83	5.72	0.95	0.10	0.38	0.16	0.10	0.08	-	-	[29]
Sichuan Deyang ^a^	90.64	5.03	1.49	0.19	1.42	0.07	0.58	0.05	0.16	9.31	[30]
Sichuan Deyang ^b^	91.03	5.47	1.28	0.20	0.63	0.05	0.31	0.12	0.11	11.64	[31]
Sichuan Chengdu	95.47	0.17	2.01	-	0.02	0.40	0.01	0.06	1.56	1.29	[32]
Anhui ^a^	88.25	8.86	0.77	0.05	0.95	0.24	0.76	0.08	-	-	[33]
Anhui ^b^	65.00	13.89	1.86	0.45	0.80	0.27	0.44	0.16	0.77	2.42	[24]
Anhui Tongling	68.97	7.14	1.25	-	0.78	-	-	-	0.67	1.87	[34]
Shandong	79.44	3.52	1.13	-	0.67	-	0.38	0.59	-	-	[35]
Shandong Binzhou	88.53	5.17	1.13	-	0.54	0.24	0.24	0.12	0.53	2.13	[36]

**Table 2:** Summary of different fluoride compounds, where - represents no literature report.

Compounds	Solubility	*K_sp_*/-	Crystallite	Ref.
NaF	Soluble	-	Y	-
KF	Soluble	-	Y	-
K_2_TiF_6_	Soluble	-	Y	-
MgF_2_ (tetragonal)	Insoluble	3.7 × 10^−8^	Y	[41]
CaF_2_	Insoluble	5.3 × 10^−9^	Y	[41]
AlF_3_	Insoluble	1.6 × 10^−33^	Y	[41]
K_2_SiF_6_	Insoluble	8.8 × 10^−4^	Y	[42]
Na_2_SiF_6_	Insoluble	1.5 × 10^−3^	Y	[42]
K_3_AlF_6_	Insoluble	2.0 × 10^−33^	Y	[41]
α-Na_3_AlF_6_(monoclinic)	Insoluble	4.0 × 10^−10^	Y	[43]
Ca_5_(PO_4_)_3_F	Insoluble	2.8 × 10^−61^	Y	[41]

**Table 3:** Summary of Miller Indices of fluoride compounds reported in the literature.

Compounds	2θ(hkl)/°			Reference
NaF	38.56(020)	55.69(022)	69.78(222)	[51,52,53]
KF	28.91(111)	33.51(020)	48.12(022)	[54]
K_2_TiF_6_	26.19(011)	31.17(110)	41.15(201)	[55,56]
MgF_2_ (tetragonal)	27.41(110)	40.62(111)	53.79(211)	[57,58,59]
CaF_2_	29.35(111)	48.89(022)	58.06(131)	[51,53,60,61,62,63]
AlF_3_	25.32(012)	42.72(113)	52.00(024)	[53,64,65,66]
K_2_SiF_6_	18.88(111)	31.07(022)	38.30(222)	[67]
Na_2_SiF_6_	20.10(110)	21.14(101)	26.82(111)	[51]
K_3_AlF_6_	18.18(134)	29.86(428)	29.97(260)	[68]
α-Na_3_AlF_6_ (monoclinic)	32.52(111)	32.68(113)	46.70(222)	[69]
Ca_5_(PO_4_)_3_F	25.81(002)	31.84(121)	33.01(030)	[70]

**Table 4:** Summary of reported binding energies and corresponding total energies estimated from DFT calculation (# refers to total energy—TE/per atom).

Compounds	Binding Energy from XPS/eV		F 1s/eV	C 1s/eV	Ref.	DFT-TE^#^/kcal/mol/atom
NaF	Na 1s (1071.50)		684.60	285.0	[71,72]	0.053
KF	K 2p_3/2_ (293.10)		684.30	285.0	[72,73]	0.261
K_2_TiF_6_	K 2p_3/2_ (293.59)	Ti 2p_1/2_(461.9)	685.10	285.0	[74]	41.886
MgF_2_ (tetragonal)	Mg 2s (89.38)		685.70	285.0	[75]	2.577
CaF_2_	Ca 2p_3/2_ (348.20)		685.00	285.0	[71,72]	8.547
AlF_3_	Al 2p_3/2_ (76.50)		688.05	285.0	[75,76]	55.227
K_2_SiF_6_	Si 2p (104.80)	K 2p_3/2_ (293.59)	687.00	285.0	[71,77]	51.625
Na_2_SiF_6_	Si 2p (104.70)	Na 1s (1071.70)	686.40	285.0	[71,77,78]	28.698
K_3_AlF_6_	Al 2p_3/2_ (75.34)	K 2p_3/2_ (293.59)	686.24	285.0	[72]	47.043
α-Na_3_AlF_6_(monoclinic)	Al 2p_3/2_ (75.97)	Na 1s (1072.23)	686.38	285.0	[75]	13.978
Ca_5_(PO_4_)_3_F	Ca 2p_3/2_ (347.7)	P 2p_3/2_ (133.60)	684.80	285.0	[71,77,79]	24.854

**Table 5:** Parametric analysis of fluoride removal using ML by physical approach, where * represents an averaged value from the datum matrix due to an absence of reports in the literature, and LSR refers to the liquid–solid ratio.

LSR/-	Times/-	F Prior to Rinse /wt%	F after Rinse/wt%	Ref.
3	3	34.0	11.0	[81]
3	4	29.0	11.0	[82]
3	3.8 *	80.0	60.0	[83]
3	5	36.0	6.0	[84]
3	6	8.0	0.0	[85]
10	1	87.0	61.0	[86]

**Table 6:** The influence of sulfuric acid treatment process parameters on the fluorine content in phosphogypsum; * refers to the values that have not been reported, where we have adopted the mean value of the array to smooth the discrete matrix for convenience of ML, and SLR refers to the solid–liquid ratio.

Concentration/%	Time/min	Temp/°C	SLR/-	F before Acid Wash/wt	F after Acid Wash/wt	Ref.
10	60	68 *	0.400	0.3000	0.1800	[94]
10	175	81	0.050	0.2031	0.0648	[95]
10	120	50	0.250	1.0600	0.1800	[96]
30	30	90	0.100	0.8700	0.2700	[86]
30	30	95	0.100	0.5630	0.0820	[97]
30	60	25	0.250	0.2600	0.1329	[98]
30	45	88	0.430	0.2030	0.0350	[95]
30	45	85	0.333	0.2700	0.0300	[99]
50	60	25	0.250	0.2600	0.0809	[98]
50	120	25	0.320 *	0.1800	0.0180	[100]

**Table 7:** Impact of operational parameters of lime treatment on fluorine content in phosphogypsum, where * refers to the values that have not been reported and the mean value of the array adopted to smooth the discrete matrix.

CaO/wt%	Duration/h	Soluble F Removal/%	Ref.
0.4	12	29.70	[100]
3	18 *	97.35	[105]
7	20 *	70.45	[106]
8	24	73.53	[81]

## Supplementary Materials

The following supporting information can be downloaded at https://www.mdpi.com/article/10.3390/ma17143606/s1. Table S1: Coordinate inputs of fluoroapatite used for spectrum estimation prior to optimization; Table S2: Optimized coordinates of fluoroapatite used for spectrum estimation via DMoI3.
