# Supplementary material for: A Review of Fluoride Removal from Phosphorous Gypsum: A Quantitative Analysis via a Machine Learning Approach
Source: Materials (Basel). 2024 Jul 22;17(14):3606. doi: 10.3390/ma17143606 (PMC11279332; doi:10.3390/ma17143606)
Supplement: Supplementary file 1 [file materials-17-03606-s001.zip › materials-3092864-supplementary.pdf]

**Table S1. Coordinates inputs of fluoroapatite used for spectrum estimation prior to optimization**

| Num | Atom | X | Y | Z |
|---|---|---|---|---|
| 1 | O | 1.997045 | 2.083177 | 0.484634 |
| 2 | O | 6.564393 | 0.687903 | 0.484634 |
| 3 | O | 5.489062 | 5.34098 | 0.484634 |
| 4 | O | 2.686455 | 6.028883 | 3.926634 |
| 5 | O | -1.88089 | 7.424157 | 3.926634 |
| 6 | O | -0.80556 | 2.77108 | 3.926634 |
| 7 | O | 2.686455 | 6.028883 | 6.399366 |
| 8 | O | -1.88089 | 7.424157 | 6.399366 |
| 9 | O | -0.80556 | 2.77108 | 6.399366 |
| 10 | O | 1.997045 | 2.083177 | 2.957366 |
| 11 | O | 6.564393 | 0.687903 | 2.957366 |
| 12 | O | 5.489062 | 5.34098 | 2.957366 |
| 13 | F | 0 | 0 | 1.721 |
| 14 | F | 0 | 0 | 5.163 |
| 15 | Ca | -2.38718 | 8.054462 | 1.721 |
| 16 | P | 2.001726 | 2.991729 | 1.721 |
| 17 | O | 0.787293 | 3.928668 | 1.721 |
| 18 | O | 3.321536 | 3.786711 | 1.721 |
| 19 | Ca | -1.09828 | 2.017468 | 1.721 |
| 20 | P | 5.775224 | 0.237681 | 1.721 |
| 21 | O | 0.887527 | 6.829542 | 1.721 |
| 22 | O | 4.426844 | 0.983179 | 1.721 |
| 23 | Ca | 3.48546 | 6.15219 | 1.721 |
| 24 | P | 6.27355 | 4.88265 | 1.721 |
| 25 | O | -1.67482 | 5.46591 | 1.721 |
| 26 | O | 6.30212 | 3.34217 | 1.721 |
| 27 | Ca | 7.070683 | 0.057598 | 5.163 |
| 28 | P | 2.681774 | 5.120331 | 5.163 |
| 29 | O | 3.896207 | 4.183392 | 5.163 |
| 30 | O | 1.361964 | 4.325349 | 5.163 |
| 31 | Ca | 5.781777 | 6.094592 | 5.163 |
| 32 | P | -1.09172 | 7.874379 | 5.163 |
| 33 | O | 3.795973 | 1.282518 | 5.163 |
| 34 | O | 0.256656 | 7.128881 | 5.163 |
| 35 | Ca | 1.19804 | 1.95987 | 5.163 |
| 36 | P | -1.59005 | 3.22941 | 5.163 |
| 37 | O | 6.35832 | 2.64615 | 5.163 |
| 38 | O | -1.61862 | 4.76989 | 5.163 |
| 39 | Ca | 0 | 5.40804 | 0.007572 |
| 40 | Ca | 4.6835 | 2.70402 | 3.449572 |
| 41 | Ca | 4.6835 | 2.70402 | 6.876428 |
| 42 | Ca | 0 | 5.40804 | 3.434428 |

**Table S2. Optimized coordinates of fluoroapatite used for spectrum estimation via DMol3.**

| Num | Atom | X | Y | Z |
|---|---|---|---|---|
| 1 | O | 3.7739 | 3.9366 | 0.9158 |
| 2 | O | 12.4049 | 1.2999 | 0.9158 |
| 3 | O | 10.3728 | 10.0930 | 0.9158 |
| 4 | O | 5.0767 | 11.3929 | 7.4203 |
| 5 | O | -3.5544 | 14.0296 | 7.4203 |
| 6 | O | -1.5223 | 5.2366 | 7.4203 |
| 7 | O | 5.0767 | 11.3929 | 12.0930 |
| 8 | O | -3.5544 | 14.0296 | 12.0930 |
| 9 | O | -1.5223 | 5.2366 | 12.0930 |
| 10 | O | 3.7739 | 3.9366 | 5.5886 |
| 11 | O | 12.4049 | 1.2999 | 5.5886 |
| 12 | O | 10.3728 | 10.0930 | 5.5886 |
| 13 | F | 0.0000 | 0.0000 | 3.2522 |
| 14 | F | 0.0000 | 0.0000 | 9.7567 |
| 15 | Ca | -4.5111 | 15.2207 | 3.2522 |
| 16 | P | 3.7827 | 5.6535 | 3.2522 |
| 17 | O | 1.4878 | 7.4241 | 3.2522 |
| 18 | O | 6.2768 | 7.1558 | 3.2522 |
| 19 | Ca | -2.0754 | 3.8125 | 3.2522 |
| 20 | P | 10.9136 | 0.4492 | 3.2522 |
| 21 | O | 10.5277 | -2.4236 | 3.2522 |
| 22 | O | 8.3655 | 1.8579 | 3.2522 |
| 23 | Ca | 6.5866 | 11.6260 | 3.2522 |
| 24 | P | 11.8553 | 9.2269 | 3.2522 |
| 25 | O | 14.5361 | 10.3291 | 3.2522 |
| 26 | O | 11.9093 | 6.3158 | 3.2522 |
| 27 | Ca | 13.3617 | 0.1088 | 9.7567 |
| 28 | P | 5.0678 | 9.6760 | 9.7567 |
| 29 | O | 7.3628 | 7.9055 | 9.7567 |
| 30 | O | 2.5737 | 8.1737 | 9.7567 |
| 31 | Ca | 10.9260 | 11.5171 | 9.7567 |
| 32 | P | -2.0631 | 14.8804 | 9.7567 |
| 33 | O | -1.6772 | 17.7532 | 9.7567 |
| 34 | O | 0.4850 | 13.4716 | 9.7567 |
| 35 | Ca | 2.2640 | 3.7036 | 9.7567 |
| 36 | P | -3.0048 | 6.1027 | 9.7567 |
| 37 | O | -5.6856 | 5.0005 | 9.7567 |
| 38 | O | -3.0587 | 9.0138 | 9.7567 |
| 39 | Ca | 0.0000 | 10.2197 | 0.0143 |
| 40 | Ca | 8.8505 | 5.1099 | 6.5187 |
| 41 | Ca | 8.8505 | 5.1099 | 12.9946 |
| 42 | Ca | 0.0000 | 10.2197 | 6.4901 |
